# Chitosan promoting formononetin and calycosin accumulation in *Astragalus membranaceus* hairy root cultures *via* mitogen-activated protein kinase signaling cascades

**DOI:** 10.1038/s41598-019-46820-6

**Published:** 2019-07-17

**Authors:** Qing-Yan Gai, Jiao Jiao, Xin Wang, Jing Liu, Zi-Ying Wang, Yu-Jie Fu

**Affiliations:** 1 Key Laboratory of Forest Plant Ecology, Ministry of Education, Northeast Forestry University, Harbin, 150040 P.R. China; 2 Engineering Research Center of Forest Bio-Preparation, Ministry of Education, Northeast Forestry University, Harbin, 150040 P.R. China

**Keywords:** Plant biotechnology, Biotic

## Abstract

Chitosan, behaving as a potent biotic elicitor, can induce plant defense response with the consequent enhancement in phytoalexin accumulation. Accordingly, chitosan elicitation was conducted to promote the production of two phytoalexins, *i*.*e*. formononetin and calycosin (also known as health-promoting isoflavones), in *Astragalus membranaceus* hairy root cultures (AMHRCs). Compared with control, 12.45- and 6.17-fold increases in the yields of formononetin (764.19 ± 50.81 μg/g DW) and calycosin (611.53 ± 42.22 μg/g DW) were obtained in 34 day-old AMHRCs treated by 100 mg/L of chitosan for 24 h, respectively. Moreover, chitosan elicitation could cause oxidative burst that would induce the expression of genes (*MPK3* and *MPK6*) related to mitogen-activated protein kinase signaling (MAPK) cascades, which contributed to the transcriptional activation of pathogenesis-related genes (*β-1*,*3-glucanase*, *Chitinase*, and *PR-1*) and eight biosynthesis genes involved in the calycosin and formononetin pathway. Overall, the findings in this work not only highlight a feasible chitosan elicitation practice to enhance the *in vitro* production of two bioactive isoflavones for nutraceutical and food applications, but also contribute to understanding the phytoalexin biosynthesis in response to chitosan elicitation.

## Introduction

*Astragalus membranaceus*, belonging to the Leguminosae family, is an economically important medicinal and food plant mainly distributed in North China^1^. *A*. *membranaceus* root is one of the most frequently used traditional medicine (Huangqi) in East Asian for the treatment of debility, chronic illness, and spleen deficiency^2,3^. Like most of leguminous plants, *A*. *membranaceus* root naturally contains a kind of typical phytoalexins *i*.*e*. isoflavones, among which formononetin (FO) and calycosin (CA) are two representative compounds associated with versatile health-promoting benefits as diverse as antioxidant, antiviral, anti-inflammatory, anti-fatigue, estrogenic, neuroprotective, and hematopoietic activities^1,4,5^. In this respect, *A*. *membranaceus* root extracts have been well recognized as functional foods/nutraceuticals according to the U.S. Dietary Supplement Health and Education Act, which can be sold as over-the-counter dietary supplements at health food markets^6,7^.

Considering that the slow growth rate of field cultivated *A*. *membranaceus* (3–5 years) can cause the long occupation of agriculture lands, and the fluctuation of phytochemical content due to varietal, geographical, ecological, and seasonal variations always results in the unstable function of extracts, plant cell/organ culture technology has emerged as a promising method that can supersede the field cultivation of *A*. *membranaceus* for bioactive phytochemical production in short growth cycles^8^. Moreover, it is worth mentioning that plant cell/organ culture technology has been endorsed by the Food and Agriculture Organization of the United Nations as a safe and feasible tool for the production of value-added foods and health compounds^9,10^. Previously, *A*. *membranaceus* hairy root cultures (AMHRCs) have been successfully developed, which can produce the comparable yields of FO and CA as against 3 year-old field cultivated *A*. *membranaceus*^11^. However, the productivity of FO and CA is still in low levels, which can be ascribed to the comfortable microenvironment of AMHRCs with the result that the two phytoalexins (FO and CA) cannot be excessively synthesized due to the lack of external stresses. To address this, elicitation, the exogenous addition of elicitors into plant cell/organ cultures that is capable of modulating defense reactions, is the most frequently adopted strategy for the enhanced production of valuable defense phytochemicals on account of the simple usage and high efficiency^12–14^. Generally, the improvement of phytochemical yield without the significant increase in cost can make plant cell/organ culture technology more attractive from the perspective of commercial application^15,16^. In consideration of this, an essential task is the search of cost-effective elicitors that can enhance FO and CA production in AMHRCs.

Chitosan is a naturally occurring substance that can be mainly found in the abandoned exoskeleton of abundance crustaceans, which has gained worldwide attention for diverse applications in food fields (*e*.*g*., food additive, food preservative, dietary fiber, etc.) and agriculture areas (*e*.*g*., seed coating, plant disease control, soil amendment, etc.) due to its non-toxicity (the toxicity being close to salt or sugar) as well as excellent biodegradability, biocompatibility, and bioactivity^17–19^. Notably, chitosan has been well acknowledged as a potent biotic elicitor that can induce plant stress response with the consequent enhancement in phytoalexin accumulation^18,19^. In consideration of the cost and safety, it is strongly recommended to use chitosan as a non-toxic elicitor for improving FO and CA productivity in AMHRCs.

Chitosan behaving as an effective “resistance elicitor” can activate plant innate immunity and defense mechanisms involving a series of biochemical and molecular reactions, in which the enhanced expression of biosynthesis genes related to the production of phytoalexins is flagged as a characteristic event^17–19^. In our recent study, it was proven that chitosan elicitation could activate the transcription of associated biosynthesis genes thus leading to the enhancement of flavonoid accumulation in *Isatis tinctoria* L. hairy root cultures^20^. However, the action mechanism of chitosan inducing phytoalexin biosynthesis is still largely unknown. The signaling cascades *via* chitosan elicitation that results in the phytoalexin enhancement appears to take different forms, depending upon the plan species^19^. Generally, mitogen-activated protein kinase (MAPK) signaling cascades play essential roles in plant immunity and stress responses^21^. Moreover, it is reported that chitosan can activate MAPK signaling cascades (triggering the production of reactive oxygen species, inducing the expression of *MPK* genes, and boosting the transcription of defense-related genes) in *Cocos nucifera* L. and *Lycopersicon esculentum* for improving their resistances to diseases^22,23^. However, there is no study about chitosan modifying MAPK signaling cascades on the stimulation of phytoalexin biosynthesis in plant *in vitro* cultures.

Based on the foregoing, the objectives of this work were to develop a feasible chitosan elicitation practice for improving the yields of two health-promoting phytoalexins (FO and CA) in AMHRCs, and to investigate whether MAPK signaling cascades can mediate the transcriptional activation of genes related to FO and CA biosynthesis. Initially, the effect of chitosan elicitation condition on FO and CA accumulation in AMHRCs was studied for achieving their optimal yields. Afterwards, the change in oxidative status, expression of genes related to MAPK signaling cascades, and transcription of pathogenesis-related genes as well as associated genes involved in FO and CA biosynthesis pathway were analyzed for understanding the mechanism of phytoalexin production induced by chitosan. To the best of our knowledge, this is the first study regarding the application of chitosan to promote isoflavone production in AMHRCs, and the exploration of chitosan inducing phytoalexin biosynthesis from the view of MAPK signaling cascades.

## Results

### Effect of chitosan elicitation on FO and CA accumulation in AMHRCs

Generally, the success and efficiency of a given elicitation practice for improving the yields of desired phytochemicals in plant *in vitro* cultures mainly depends on the elicitor dosage and exposure time^14^. Accordingly, it is necessary to find the appropriate chitosan concentration and elicitation time for achieving the the optimal enhancement of FO and CA in AMHRCs. As reported previously, AMHRCs harvested at day 34 can give the highest productivity of isoflavone and root biomass. Thus, chitosan elicitation experiments were carried out using 34 day-old AMHRCs in this work, with the aim of further increasing FO and CA yield without affecting the biomass amount.

**Figure 1:**
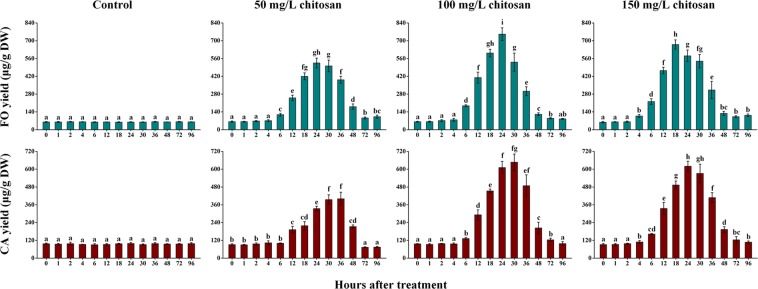
Effect of chitosan concentration (50, 100, and 150 mg/L) on the yields of FO and CA in 34 day-old AMHRCs along a 96 h time course. Results sharing different lowercase letters indicate significant differences (*P* < 0.05) between diverse groups of data.

**Figure 2:**
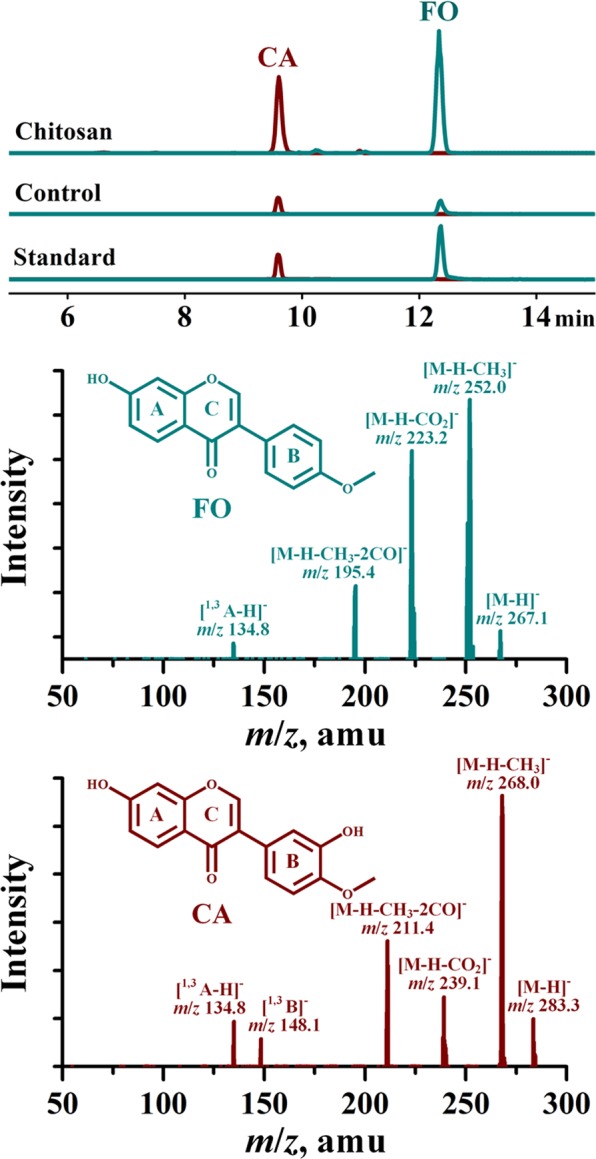
Chromatograms of FO and CA in extracts form control and chitosan-treated AMHRCs (100 mg/L and 24 h) determined by LC-MS/MS using selected reaction monitoring mode.

As exhibited in Fig. 1, chitosan elicitation using different dosages (50, 100, and 150 mg/L) showed distinct effect on the yields of two target isoflavones in AMHRCs during the time course of 0 to 96 h. Moreover, the same chitosan dosage exerted different influences on the accumulation pattern of FO and CA during the time course of experiments. This can be ascribed to the fact that the same elicitor possesses varied abilities of inducing the biosynthesis of different phytochemicals in a given plant *in vitro* culture^13^. Additionally, it was observed from Fig. 1 that chitosan could enhance FO and CA accumulation in AMHRCs after 4 h, regardless of its concentrations. This can be attributed to the rapid defense response when plant *in vitro* cultures treated by elicitors, which always cause the phytochemical profile change in a very short time^14^. Moreover, it was noted that the enhancement of FO and CA in AMHRCs was not significant during the prolonged time course of 72 to 96 h. Taken as a whole, it was found that 100 and 150 mg/L of chitosan could induce the remarkable enhancement in the yields of FO and CA during the elicitation duration of 18 to 30 h. More exactly, 100 mg/L of chitosan showed the best elicitation effect at 24 and 30 h for FO and CA, respectively. However, it is noteworthy that CA yield at 30 h was only a little higher than that at 24 h. In view of time saving, 100 mg/L of chitosan along with 24 h of exposure time were found to be appropriate for the both compounds, where the yields of FO (764.19 ± 50.81 μg/g DW) and CA (611.53 ± 42.22 μg/g DW) were 12.45- and 6.17-fold higher than those in control (61.40 ± 2.73 and 99.07 ± 6.08 μg/g DW), respectively. Moreover, the changes in LC-MS/MS chromatograms of the two target compounds in extracts form control and chitosan-treated AMHRCs (100 mg/L and 24 h) can be obviously observed in Fig. 2.

### Assessment of oxidative burst in chitosan-treated AMHRCs

**Figure 3:**
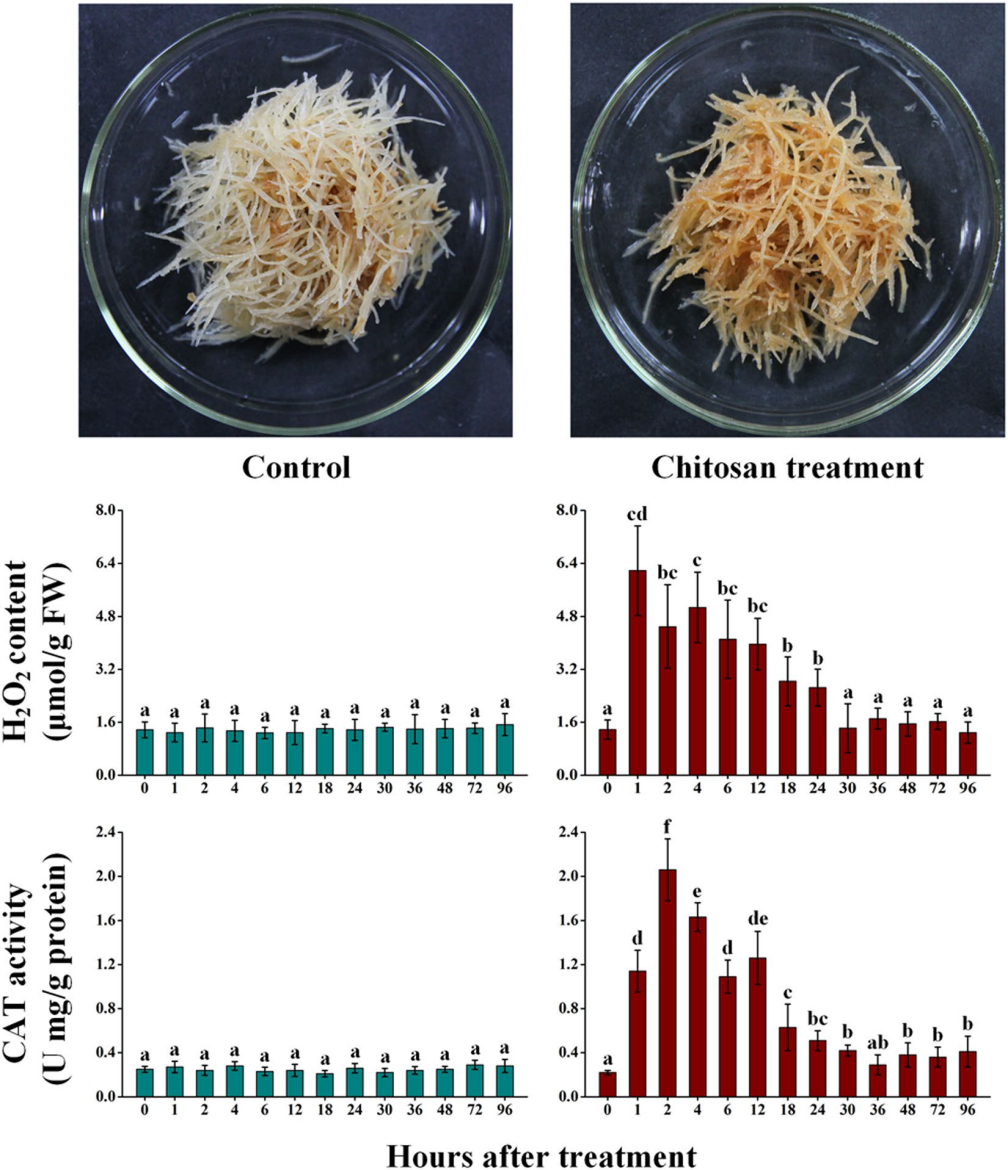
Morphological comparisons of control and chitosan-treated hairy roots (100 mg/L and 24 h); H_2_O_2_ content and CAT activity in control and chitosan-treated AMHRCs (100 mg/L) along a 96 h time course. Results sharing different lowercase letters indicate significant differences (*P* < 0.05) between diverse groups of data.

In this work, morphological comparisons of chitosan- and non-treated root cultures showed that the former one exhibited a significant indication of ROS-mediated oxidative stress (browning color) as against the later one (Fig. 3). Among all forms of ROS (O_2_^•−^, OH•, HO_2_•, H_2_O_2_, and RO•), H_2_O_2_ is considered as the most stable one, and can be accurately monitored^22,24^. Additionally, catalase (CAT) is an indispensable antioxidant enzyme that can effectively dismutate H_2_O_2_ into H_2_O and O_2_^24^. In this regard, H_2_O_2_ content and CAT activity in chitosan- and non-treated AMHRCs harvested at different time points were determined, which aimed to verify the oxidative burst whether occur in AMHRCs following chitosan elicitation. In comparison with non-treated control, H_2_O_2_ content in chitosan-treated AMHRCs increased immediately, reached the peak value (6.19 ± 1.35 μmol/g FW) at 1 h, and decreased slowly to the control level afterwards (Fig. 3). Moreover, CAT activity was noticed to increase very rapidly, achieve the highest value (2.06 ± 0.28 U mg/g protein) at 2 h, and declined gradually to a stable level subsequently (Fig. 3). Obviously, the instantaneous increase of H_2_O_2_ lead to the fast enhancement in CAT activity during the early elicitation period, followed by the consumption of antioxidant enzyme accompanied by the decrease in H_2_O_2_ content during the late elicitation period, which indicated a positive-feedback response to fight the oxidative stress mediated by ROS.

### MAPK gene expression in chitosan-treated AMHRCs

**Figure 4:**
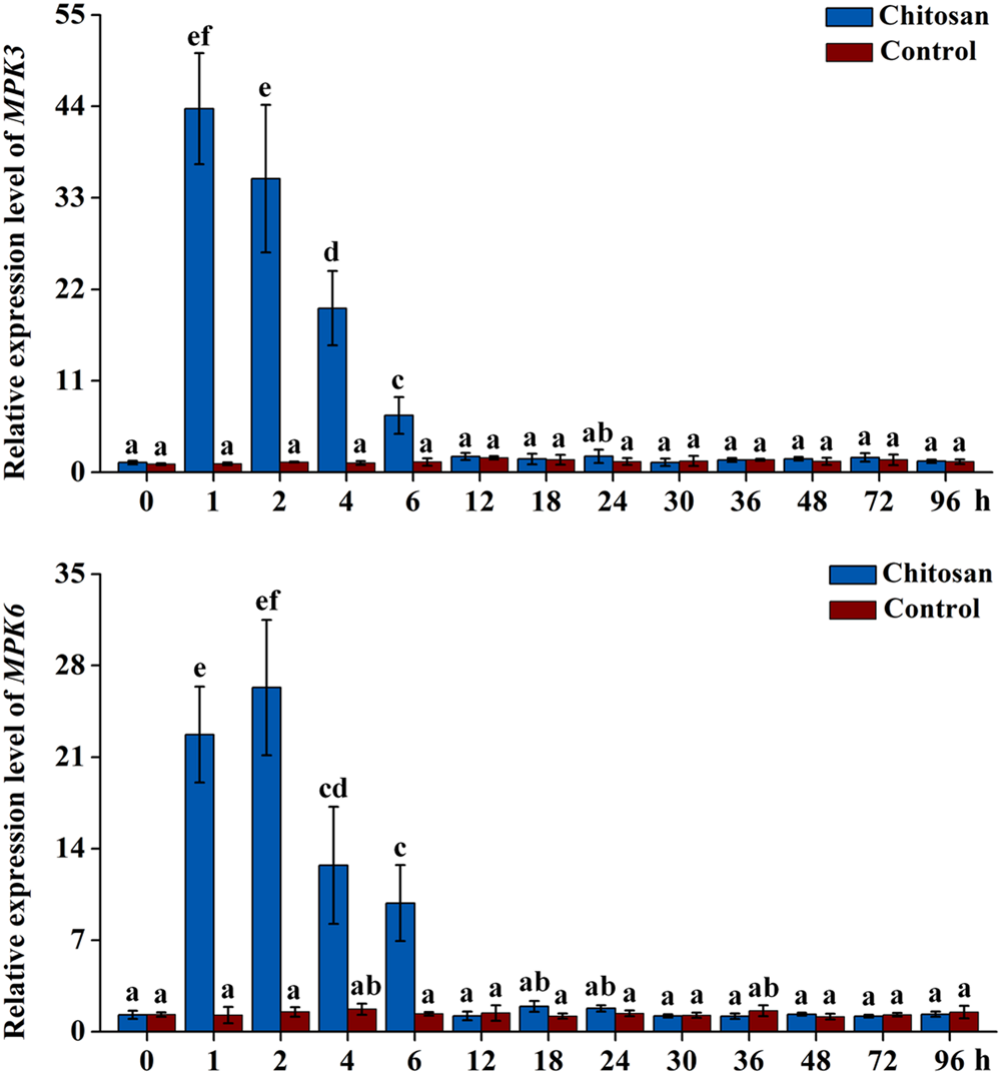
Transcriptional levels of *MPK3* and *MPK6* in AMHRCs treated with chitosan (100 mg/L) along a 96 h time course. Results sharing different lowercase letters indicate significant differences (*P* < 0.05) between diverse groups of data.

Factually, MAPK cascade-mediated signalling is quite essential in the regulation of many biological processes in plants, such as growth, development, and programmed cell death, and especially in immunity and stress responses^21,25^. Two well-characterized MAPKs, *i*.*e*. MPK3 and MPK6, are regarded as key mediators that can positively regulate various defense signaling in response to a diversity of biotic or abiotic stimuli^25^. More importantly, it is reported that MPK3/MPK6 cascade play an integral role in phytoalexin biosynthesis for the defense against fungal pathogen attacks^26^. To verify whether MPK3/MPK6 cascade can be activated by ROS in chitosan-treated AMHRCs, the corresponding gene transcripts along the time course of 0–96 h were determined by qRT-PCR. As shown in Fig. 4, the transcriptional level of *MPK3* in AMHRCs was induced immediately after chitosan treatment, reached the highest level at 1 h (43.72-fold increase), and reduced rapidly to the basal level after 6 h. Meanwhile, *MPK6* expression was also observed to respond rapidly following chitosan treatment, achieve the peak level (26.31-fold increase) at 2 h, and decrease significantly to the control level after 6 h (Fig. 4). As expected, the transient transcription of both genes indicated that the MPK3/MPK6 cascade was indeed activated by ROS in chitosan-treated AMHRCs.

### Pathogenesis-related (PR) gene expression in chitosan-treated AMHRCs

**Figure 5:**
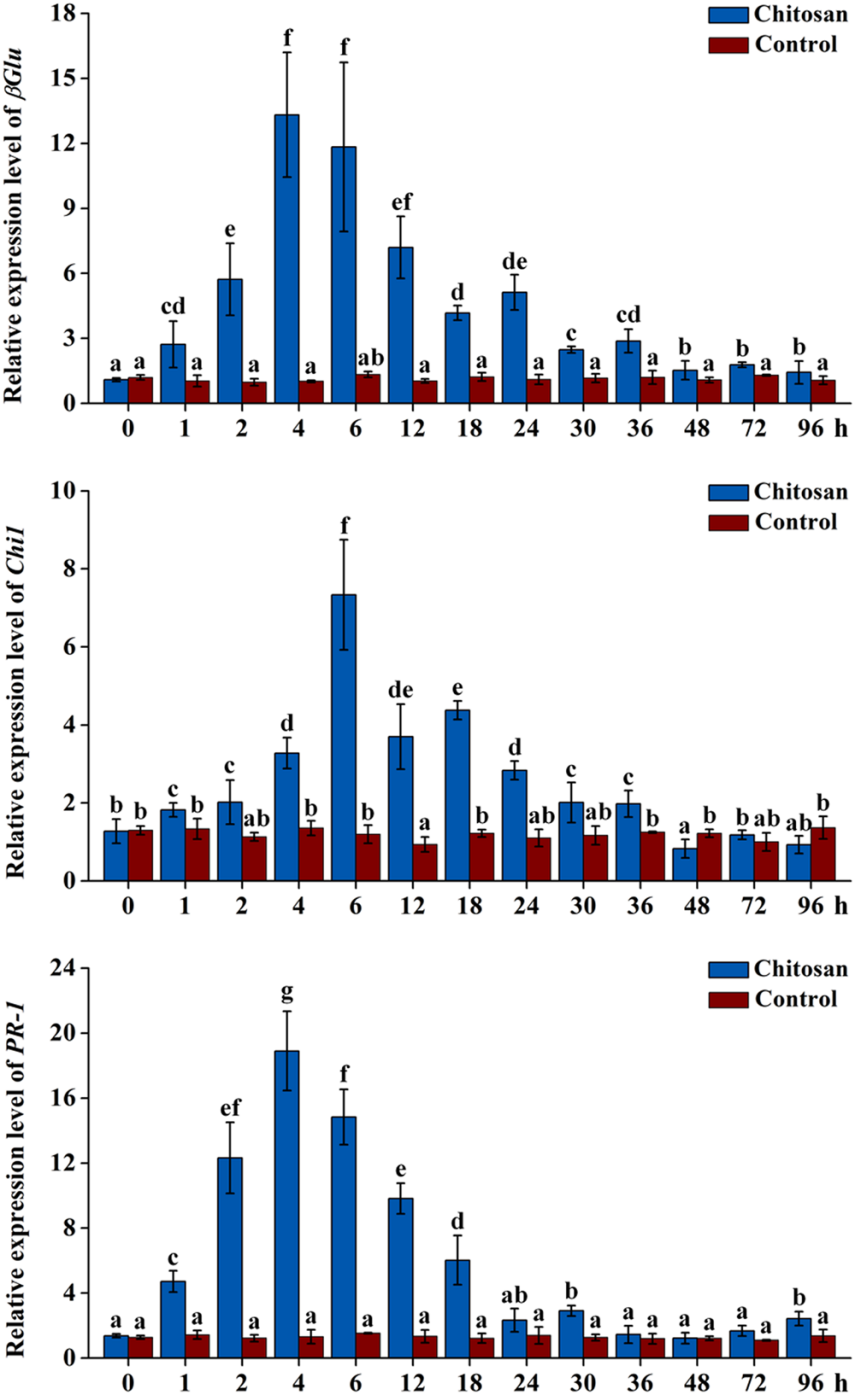
Expression levels of *βGlu*, *Chi1*, and *PR-1* in AMHRCs treated with chitosan (100 mg/L) along a 96 h time course. Results sharing different lowercase letters indicate significant differences (*P* < 0.05) between diverse groups of data.

The major actions of MAPK cascade after being activated is to relocate to the nucleus where they can induce the expression of defense-related genes through activation of specific transcription factors^23,25^. In this study, *β-1*,*3-glucanase* (*βGlu*), *class 1 chitinase* (*Chi1*), *pathogen-related protein 1* (*PR-1*) were selected as marker defense genes to detect if they can be induced by the MPK3/MPK6 cascade in chitosan-treated AMHRCs. Also, the relative expression levels of these genes along the time course of 0–96 h were determined by qRT-PCR. It was noticed from Fig. 5 that *βGlu* expression was gradually induced to the highest level (13.32-fold increase) at 4 h, and decreased slowly afterwards. And, the transcriptional level of *Chi1* was found to be highest (7.70-fold increase) at 6 h, after which a gradual decline tendency was observed (Fig. 5). Moreover, the significant induction of *PR-1* expression (9.93 to 18.89-fold increase) was found during the time course from 2 to 12 h, whereas *PR-1* transcription reduced to the value around the basal level after 18 h (Fig. 5). Obviously, chitosan treatment led to an enhanced expression of *βGlu*, *Chi1*, and *PR-1* in a very short time.

### Biosynthesis genes expression in chitosan-treated AMHRCs

**Figure 6:**
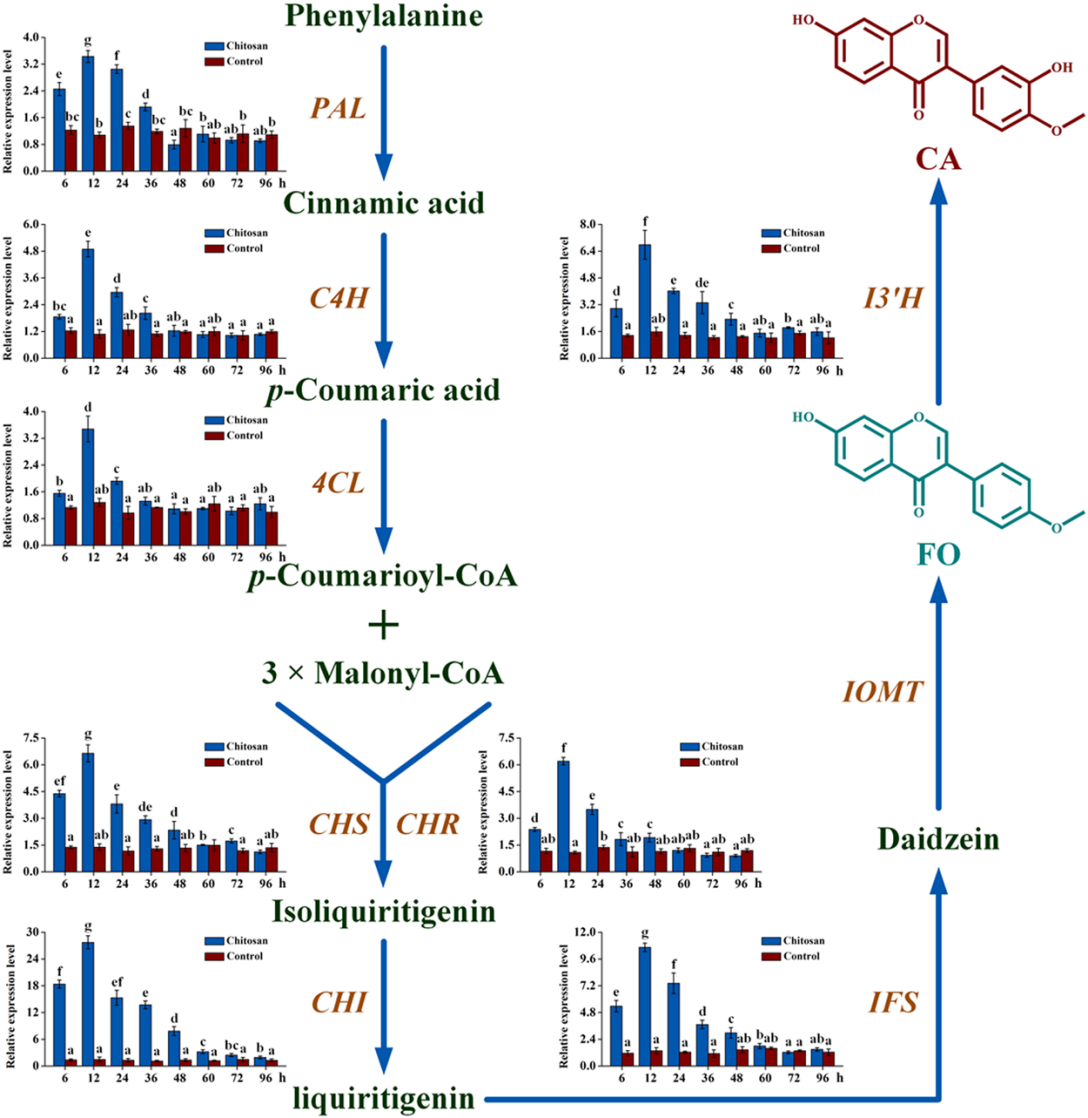
Transcriptional profiles of eight biosynthesis genes involved in FO and CA pathway in AMHRCs treated with chitosan (100 mg/L) from 6 to 96 h. Results sharing different lowercase letters indicate significant differences (*P* < 0.05) between diverse groups of data.

Transcriptional profiles of *phenylalanine ammonia lyase* (*PAL*), *cinnamate*-*4*-*hydroxylase* (*C4H*), *4*-*coumarate coenzyme A ligase* (*4CL*), *chalcone synthase* (*CHS*), *chalcone reductase* (*CHR*), *chalcone isomerase* (*CHI*), *isoflavone synthase* (*IFS*), *and isoflavone 3*′-*hydroxylase* (*I3*′*H*) that are involved in FO and CA biosynthesis pathway along the time course of experiments (6–96 h) were determined by qRT-PCR, which aimed to test whether MAPK cascade-mediated signal transduction can up-regulate the aforementioned genes in AMHRCs underlying chitosan treatment. As shown in Fig. 6, the transcriptional levels of all tested genes were significantly up-regulated in chitosan-treated AMHRCs during the period from 6 to 36 h, which indicated that MAPK-mediated signal transduction could indeed positively regulate the expression of these biosynthesis genes, thus contributing to the enhanced accumulation of FO and CA along the same time course (6 to 36 h). Taken as a whole, the transcriptional profiles of all target genes were almost consistent (Fig. 6). Their expression increased up to the highest level from 6 to 12 h, and decreased gradually to the basal level in the following experimental period (24 to 96 h). Additionally, it is noteworthy that the time point (12 h) required the highest expression levels of all biosynthesis genes was earlier than that (24 h) necessary for the maximal accumulation of FO and CA (Fig. 6), which can be attributed to a typical metabolic phenomenon that the downstream product synthesis lagged behind the upstream gene expression^27^. Moreover, *CHI* exhibited the highest expression abundances (27.72-fold) at 12 h among all tested genes (Fig. 6).

## Discussion

Generally, isoflavones are considered as phytoalexins in leguminous plants that are strongly inducible and especially sensitive to pathogen and insect challenges^28^. It is known that chitin is a typical pathogen-associated molecule present in fungal cell walls^22^. Thus, chitosan (the derivative of chitin) is able to elicit plant defense response *via* simulating the attacks of fungal pathogen, which can eventually result in the enhancement of phytoalexin biosynthesis^17^. In this regard, it is not surprising that chitosan elicitation can promote FO and CA biosynthesis in AMHRCs in this work (Fig. 1).

Chitosan elicitation is reported to possess positive influence on promoting secondary metabolite accumulation in many plant *in vitro* cultures, such as echinacoside in *Scrophularia striata* Boiss. cell suspension cultures^29^, hydrolysable tannin in *Phyllanthus debilis* Klein ex Willd. cell suspension cultures^30^, plumbagin in *Plumbago indica* L. adventitious root cultures^31^, and withanolides in *Withania somnifera* (L.) Dunal adventitious root cultures^32^. Whereas, chitosan elicitation conditions required for the maximal enhancement of the abovementioned phytochemicals are inconsistent. Factually, plants exposed to elicitation/stress have tolerance-limit levels for activating defense secondary metabolisms and possess thresholds for biosynthesizing specific secondary metabolites, which depends upon the inherent property of plant species^14^. Moreover, when secondary metabolites are accumulated excessively, plants can be induced to generate special enzymes for the degradation of target metabolites *via* the feedback regulation^15^. Overall, it is inferred that 34 day-old AMHRCs treated by chitosan (100 mg/L and 24 h) (Fig. 1) might suffer from the appropriate stress for inducing FO and CA biosynthesis without degradation.

Generally, the action mechanism of elicitor inducing secondary metabolic responses in plant cells is complex owing to the existence of thousands of intertwined events^13,14^. The action of elicitors can alter plant cellular activities at biochemical and molecular levels, which usually triggers multiple signal transduction cascades that ultimately activate the transcription of enzymatic genes involved in the biosynthesis pathways of defense secondary metabolites^12–14^. In this regard, it is important to understand the action mechanism of chitosan inducing the biosynthesis of FO and CA.

As mentioned above, chitosan elicitation is able to simulate fungal pathogen attacks. This can be recognized by cell surface-located pathogen-recognition receptors, and cause the oxidative burst (one of the earliest cellular responses), *i*.*e*. the over-production of highly toxic reactive oxygen species (ROS)^22,29^. On one hand, ROS can activate antioxidant enzymes to scavenge them for detoxifying the oxidative damages to membranes, nucleic acids, lipids, and proteins in plant cells^24^. On the other hand, ROS can act as signal molecules, trigger many signal transduction cascades, and eventually cause the enhanced expression of specific genes related to the production of phytoalexins with antioxidant properties, which can neutralize ROS for maintaining the cellular redox status in plant cells^33,34^. Results in this work (Fig. 3) confirmed the actual occurrence of oxidative burst in AMHRCs undergone chitosan elicitation, which might trigger some special signaling cascades that contributed to activating the transcription of genes related to FO and CA biosynthesis.

As described previously, ROS functions as a general priming signal that can contribute to triggering multiple intracellular signalling cascades in plants, among which the activation of MAPK signaling pathways is considered as a common event^33,36^. Generally, external stressors/elicitors can trigger plant immunity requiring a signal transduction from receptors to downstream defense components^35^. Thus, the rapid and strong activation of MPK3/MPK6 cascade in chitosan-treated AMHRCs (Fig. 4) might contribute to transducing stress signals to downstream components for the enhanced expression of genes related to the production of defense-related molecules.

Generally, the activation of MAPK cascade is followed by the induction of PR genes that can encode PR proteins (*e*.*g*., *β*-1,3-glucanase, chitinase1, and PR-1 protein), which are major components of downstream immune signaling and consequently play a critical role in plant defense systems^22,25,36^. It is known that the upregulation of PR genes is correlated with the onset of plant immune response, known as systemic acquired resistance (SAR), which can make plants becomes more resistant to pathogens^22,37^. So, the synergistic activation of three PR genes in this work (Fig. 5) indicated that chitosan-induced stress signals can be successfully transduced *via* the MAPK cascade, thus leading to a typical SAR response in chitosan-treated AMHRCs for the enhanced resistance.

In addition to triggering PR gene expression, the activation of enzymatic genes related to phytoalexin biosynthesis is considered as a common event after MAPK cascade relaying the defense signals to specific transcription factors in nucleus^25,26^. In this work, the synergistic expression tendency of all biosynthesis genes along the time course of experiments (Fig. 6) was in accordance with the typical rule that transcription factors can activate the transcription of multiple target genes^38^. Also, this confirmed that MAPK cascade relayed the signals to specific transcription factors that simultaneously regulated the expression of genes involved in FO and CA biosynthesis pathway. Moreover, the highest expression abundances (27.72-fold) of *CHI* at 12 h among all tested genes (Fig. 6) suggested that this gene might be most susceptible to chitosan treatment for promoting FO and CA biosynthesis in AMHRCs.

Factually, CHI is capable of catalyzing the intramolecular cyclization of bicyclic chalcone to form tricyclic (2S)-flavanone, which is well recognized as the rate-limiting step controlling the downstream flavonoid metabolism^39^. Accordingly, CHI is a key enzyme that can effectively regulate flavonoid biosynthesis. CHI has been verified as a unique enhancer to control flavonoid biosynthesis in *Arabidopsis thaliana*^40^. And, the expression of *CHI* gene was found to correlate positively with flavonoid accumulation in *Lycium chinense*, *Citrus unshiu*, and *Ipomoea batatas* (L.) Lam^41–43^. Moreover, the overexpression of *CHI* gene led to a 78-fold increase in flavonol level in the transgenic tomato^44^. Also, the overexpression of *CHI* gene resulted in the transgenic *Scutellaria baicalensis* hairy roots containing the maximum 2.89- and 8.82-fold increase in the yields of baicalein and wogonin, respectively^45^. Additionally, the overexpression of *CHI* gene caused the enhanced accumulation of daidzein in the transgenic soybean^46^. In this study, the significant increase in the productivity of FO (12.45-fold increase) and CA (6.17-fold increase) in chitosan-treated AMHRCs might be partially ascribed to the remarkable up-regulation of *CHI* expression. However, the weakest up-regulated genes might also cause the enhanced biosynthesis of secondary metabolites in plants. Therefore, it was inferred that the synergistic effects of all up-regulated biosynthesis genes contributed to the significant improvement in FO and CA yields.

**Figure 7:**
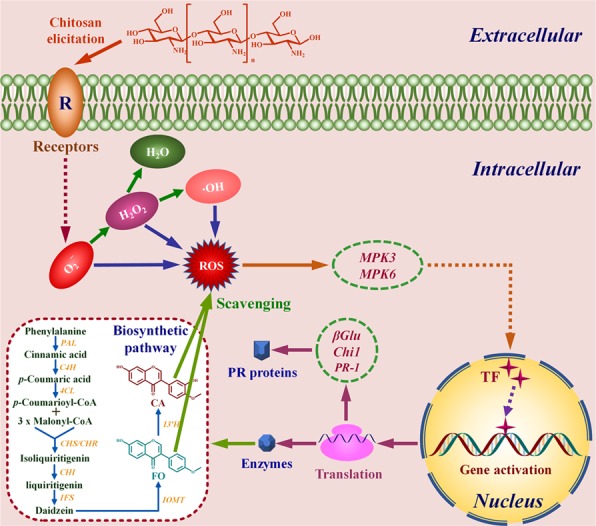
The putative schematic diagram of chitosan-induced FO and CA biosynthesis in AMHRCs *via* MAPK signaling cascades. The dotted lines indicate the processes with no detailed evidence provided in this study.

Based on the above studies, the induction of FO and CA biosynthesis in AMHRCs *via* chitosan-mediated MAPK signaling cascades is illustrated schematically in Fig. 7. In detail, chitosan elicitation was initially perceived by the special receptors present on plant cell membranes, which would cause the alteration of ion efflux/influx (need to be further verified), thus resulting in the massive production of ROS. Subsequently, ROS acted as a priming signal to activate the MPK3/MPK6 cascade, which relayed the stress signaling to specific transcription factors (need to be further verified) in nucleus. After that, PR genes (e.g., *βGlu*, *Chi1*, and *PR-1*) were activated to enhance PR protein synthesis for improving the resistance of plant cell itself. Moreover, the specific transcription factors in nucleus after being activated was to up-regulate the expression of biosynthesis genes for promoting the production of two isoflavone phytoalexins (FO and CA), both of which could scavenge the excessive ROS for restoring the intracellular redox status in equilibrium.

In conclusion, a simple and feasible elicitation practice using chitosan was proposed in this work for improving the yield of two health-promoting phytoalexins (FO and CA) in AMHRCs. The low cost, nontoxicity and biocompatibility of chitosan will make the proposed elicitation protocol more commercially attractive for the scale-up production of the two bioactive isoflavones. More importantly, this study preliminarily elucidated the molecular mechanisms that the enhanced biosynthesis of FO and CA in AMHRCs was achieved by chitosan-mediated MAPK signaling cascades. However, further studies need to be done to identify the specific MAPK or the upstream kinase of MPK3/MPK6 induced by chitosan in AMHRCs.

## Methods

### Establishment and maintenance of AMHRCs

In our previous study, *A*. *membranaceus* hairy roots were established *via Agrobacterium rhizogenes* LBA9402 mediated transformation of leaf explants^11^. A hairy root line (AMHRL II) stocked in our laboratory with the high productivity of isoflavone and biomass was adopted for the subsequent experiments. Moreover, AMHRCs used in this work were obtained by cultivating AMHRL II under the optimized conditions reported by Jiao *et al*.^11^.

### Treatment of AMHRCs by chitosan

The chitosan stock solution (10 mg/mL) was prepared using acetic acid (pH = 5.5), and autoclaved prior to use. In this work, 34 day-old AMHRCs (the optimal cultures for harvesting hairy roots) would undergo chitosan elicitation with different dosage and exposure time. Before elicitation, the fresh media were used to replace the spent ones in AMHRCs, and the chitosan stock solution was added into the renewed AMHRCs to give different final concentrations (50, 100, and 150 mg/L). In addition, AMHRCs treated with the equal volume of acetic acid solution were defined as control cultures. Subsequently, all tested AMHRCs were cultured on a gyratory shaker (120 rpm) at 25 ± 1 °C without light, and sampling was performed at different time points (0, 1, 2, 4, 6, 12, 18, 24, 30, 36, 48, 72, and 96 h). After elicitation, the collected hairy roots were rinsed thoroughly with tap water and distilled water, and divided into three parts: one being directly used for the valuation of oxidative burst, one being frozen immediately using liquid nitrogen for RNA extraction, and the rest one being dried completely for isoflavone extraction.

### LC-MS/MS analysis

The isoflavone extraction from dried hairy root powders and sample preparation for LC-MS/MS analysis were performed using the methods reported by Jiao *et al*.^11^. Two target isoflavones (FO and CA) in tested samples were analyzed using the established LC-MS/MS method reported by Jiao *et al*.^11^. Two ion combinations of precursor ion/product, *i*.*e*. *m/z* 267.0 → 252.0 and *m/z* 283.0 → 268.0, were used to determine FO and CA in the extracts form root samples, respectively. The content of each target compound was expressed as microgram per gram of the dry weight (DW) of root samples.

### Evaluation of oxidative burst

The level of H_2_O_2_ in fresh hairy root samples was measured according to the protocols described by Dewanjee *et al*.^47^, and the amount was expressed as micromole per gram of the fresh weight (FW) of root samples. Additionally, the activity of CAT in fresh hairy root samples was determined as the descriptions of Arbona *et al*.^48^, and the value was expressed as units per microgram of protein that was detected in enzyme extracts.

### qRT-PCR analyses

To study the action mechanism of chitosan inducing phytoalexin biosynthesis, the transcription levels of genes related to MAPK signaling cascades, pathogenesis-related genes, and biosynthesis genes involved in FO and CA pathway were tested by qRT-PCR, *i*.*e*. *MPK3*, *MPK6*, *βGlu*, *Chi1*, *PR-1*, *PAL*, *C4H*, *4CL*, *CHS*, *CHR*, *CHI*, *IFS* and *I3*′*H*. Specific primers of genes (*MPK3*, *MPK6*, *βGlu*, *Chi1*, and *PR-1*) related to MAPK signaling cascades (Table 1) were designed based on the transcriptome sequences reported by Liu *et al*.^49^. Primers of genes (*PAL*, *C4H*, *4CL*, *CHS*, *CHR*, *CHI*, *IFS*, and *I3*′*H*) associated with FO and CA biosynthesis (Table 1) were adopted as described previously^50^. RNA Extraction, reverse-transcription, and gene amplification were performed using the methods reported by Jiao *et al*.^50^. The relative transcription levels of all tested genes (*MPK3*, *MPK6*, *βGlu*, *Chi1*, *PR-1*, *PAL*, *C4H*, *4CL*, *CHS*, *CHR*, *CHI*, *IFS* and *I3*′*H*) were calculated based on the internal reference 18*S* gene using the method reported by Livak and Schmittgen^51^.

**Table 1:** Primers of MAPK signaling cascade genes, pathogenesis-related genes, and biosynthesis genes involved in the FO and CA pathway.

Gene names	Primer sequences (5′ to 3′)	Product sizes (bp)
*MPK3*	Forward: TGAGGTGAAGTTCAACGTGAGGTC	167
	Reverse: CTGGTGGAAGAGGCAATGGATGAC	
*MPK6*	Forward: TGCTATGCTGGCAAGCTCAATGG	147
	Reverse: CCTATGGCACACTCAAGCACTACC	
*βGlu*	Forward: TCCGATTGCCGAGCAGAATTGG	124
	Reverse: CGATTGTCCTCCTCCTCCTCCTC	
*Chi1*	Forward: CCAGTCACCTAAGCCATCTTGCC	153
	Reverse: ACTCTTCCATCCTGTCCTCTTCCG	
*PR-1*	Forward: GCGTAACGACTGTGCCTTGGAG	98
	Reverse: CACTTACTGCCTGTGCTGGATTCC	
*PAL*	Forward: CATCAAATCTCTCTGGCAGTAGGAA	147
	Reverse: AGTTCACATCTTGGTTATGCTGCTC	
*C4H*	Forward: AACAAAGTGAGGGATGAAATTGACA	127
	Reverse: GGATTGCCATTCTTAGCCTTAGTGT	
*4CL*	Forward: TGTCCCTCCTATTGTTTTGGCTATT	140
	Reverse: CTTTGGGGAATTTAGCTCTGACAGT	
*CHS*	Forward: CCTTCTTTGGATGCTAGACAAGACA	188
	Reverse: CGAAGACCCAAGAGTTTGGTTAGTT	
*CHR*	Forward: AAACAAGGTTACAGGCATTTTGACA	164
	Reverse: GGAAGAACGAGATGAGGATGATTTT	
*CHI*	Forward: ATCGAGTTTTTCCACCAGGATCTAC	154
	Reverse: ATCATAGTCTCCAACACAGCCTCAG	
*IFS*	Forward: CCTTCACCTATTGGACAAACCTCTT	172
	Reverse: CCTGGTATTAAAGGAAGAAGCCTCA	
*I3*′*H*	Forward: GGATGTTAAAGAAGCGAAGCAATTT	106
	Reverse: ATCAAACAATCTCAACAAAGGCAAA	
*18S*	Forward: TGCAGAATCCCGTGAACCATC	104
	Reverse: AGGCATCGGGCAACGATATG	

### Statistical analyses

Results were obtained from the triplicate experiments, and reported as averages ± standard deviations. The SPSS statistical software (version 17.0, SPSS Inc, USA) was used to carry out all statistical analyses. One-way analysis of variance using Tukey’s test was applied to determine the significant differences (*P* < 0.05) between diverse groups of data.

## Data Availability

All data generated or analyzed during this study are included in this manuscript.
